# Effectiveness of Extracorporeal Shock Wave Therapy in Calcinosis of Dermatomyositis: A Case Report

**DOI:** 10.7759/cureus.116144

**Published:** 2026-09-12

**Authors:** Najia Hajjioui, Hanane Baybay, Fatima Zahra Mernissi, Abderrazak Hajjioui

**Affiliations:** 1 Faculty of Medicine and Pharmacy, Sidi Mohamed Ben Abdellah University, Fez, MAR; 2 Department of Physical Medicine and Rehabilitation, University Hospital Hassan II, Fez, MAR; 3 Department of Dermatology, University Hospital Hassan II, Fez, MAR; 4 Department of Physical and Rehabilitation Medicine, Faculty of Medicine and Pharmacy, Abdelmalek Essaâdi University, Tangier, MAR

**Keywords:** calcinosis, case report, dermatomyositis, extracorporeal shockwave therapy, rehabilitation

## Abstract

Dermatomyositis is an autoimmune connective tissue disease, in which calcinosis represents a late and often disabling complication, particularly in the juvenile form. The therapeutic management of calcinosis includes medical, surgical, and physiotherapy approaches, but no standardized treatment has been established. Extracorporeal shock wave therapy (ESWT) has emerged as a potential therapeutic alternative; however, reports of ESWT use in calcinosis associated with dermatomyositis remain rare.

We report a case of dermatomyositis-associated calcinosis treated with ESWT, with favorable clinical outcomes, suggesting a potential therapeutic role for this approach in this challenging condition.

## Introduction

Cutaneous calcinosis is the abnormal deposition of insoluble calcium salts in tissues, including skin, subcutaneous tissue, tendons, fascia, and muscle. Calcinosis is more commonly seen in juvenile dermatomyositis (JDM), which is a rare idiopathic inflammatory myopathy characterized by muscle weakness and cutaneous manifestations in children [1]. The functional impact can be significant, especially when the calcinosis is located around a joint and has repercussions on psychosocial and school life, hence the need for medical and psychosocial care. Calcinosis in JDM is a form of dystrophic calcification that occurs in damaged or chronically inflamed tissues, generally despite normal serum calcium and phosphate levels. It may develop months to years after disease onset and may persist even after control of the underlying inflammatory disease. Its course is variable, ranging from stable or regressive lesions to progressive deposits causing pain, ulceration, secondary infection, joint contractures, and functional impairment.

Several treatments have been proposed: warfarin, colchicine, bisphosphonates, diltiazem, aluminium hydroxide, corticosteroid injections, surgery and physiotherapy [1]. Physiotherapy includes range-of-motion and stretching exercises, progressive muscle strengthening, and functional rehabilitation to maintain mobility and prevent contractures. However, the treatment regimen and administration modalities remain a challenge for the practitioner, as no specific treatment has been recommended for calcinosis in patients with juvenile calcific dermatomyositis [2]. Extracorporeal shock wave therapy (ESWT) has been reported as a potential therapeutic option for calcinosis, although evidence remains limited.

The objective of this case report is to describe changes in calcinosis area and pain over 24 months following ESWT in a patient with JDM.

## Case presentation

A 15-year-old patient, without any particular medical history, a student in the first year of high school, and the eldest of 4 siblings, was referred by dermatologists to the physical medicine and rehabilitation department for non-pharmacological management of progressively worsening cutaneous calcifications in the context of JDM evolving since the age of 10 years. The patient was initially put on corticosteroids, at a dose of 2 mg/kg/day. This was followed by immunosuppressive drugs, several courses of intravenous immunoglobulin, and calcium channel blockers in the later stages, after the onset of cutaneous calcinosis.

Two years after the onset of his disease, the clinical (Figure 1) and radiological evolution (Figure 2) were unfavorable. The progressive appearance of areas of cutaneous calcinosis, including the initial appearance of calcinosis in his left arm and right popliteal fossa with a secondary flexor of 45° in the context of six months of treatment discontinuation by the parents, was noted. ESWT therapy with functional rehabilitation was proposed.

**Figure 1:**
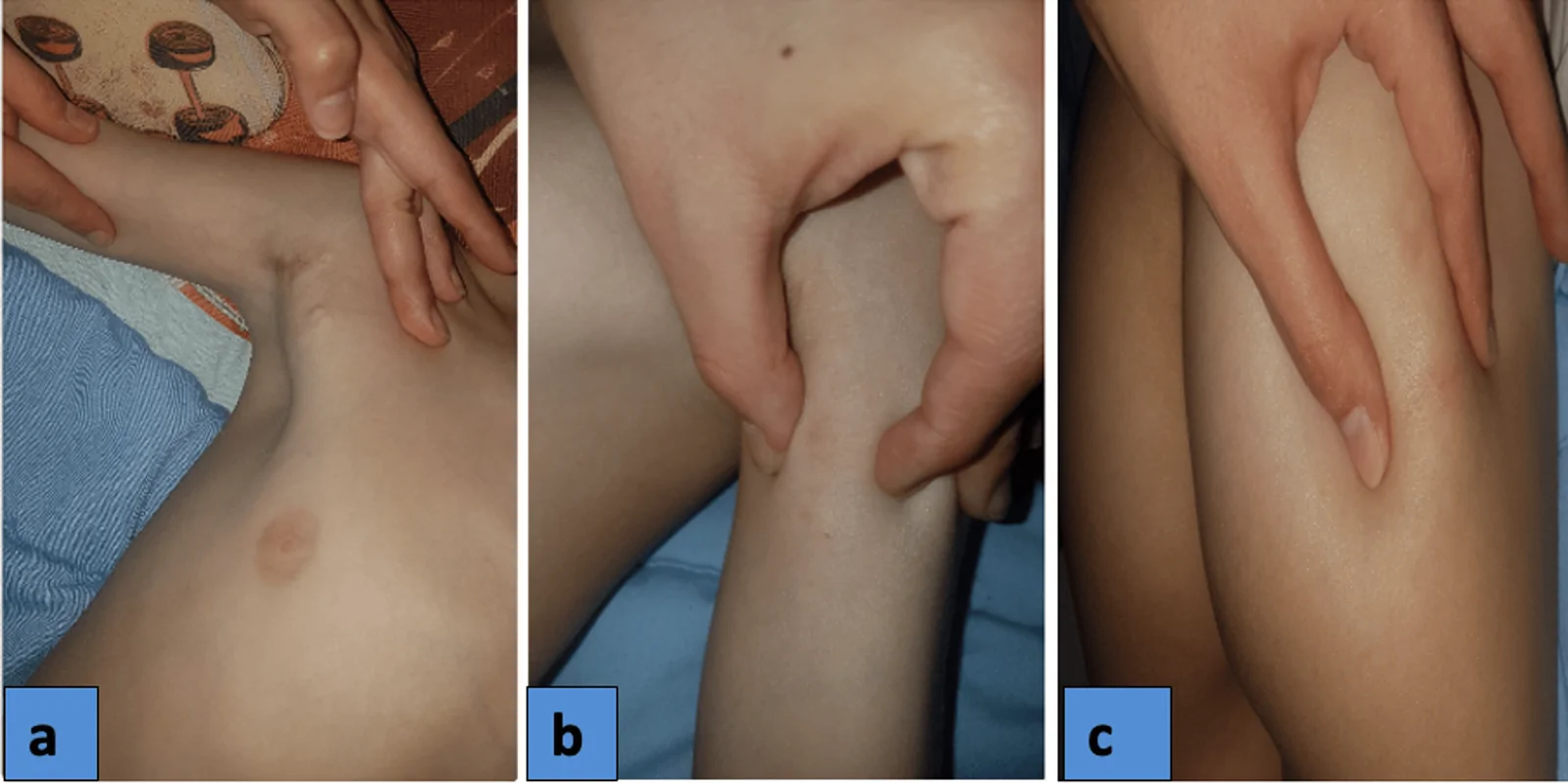
Calcinosis in a patient with juvenile dermatomyositis before ESWT treatment Calcinosis in the upper limbs (a,b) and lower limb (c) ESWT: extracorporeal shock wave therapy

**Figure 2:**
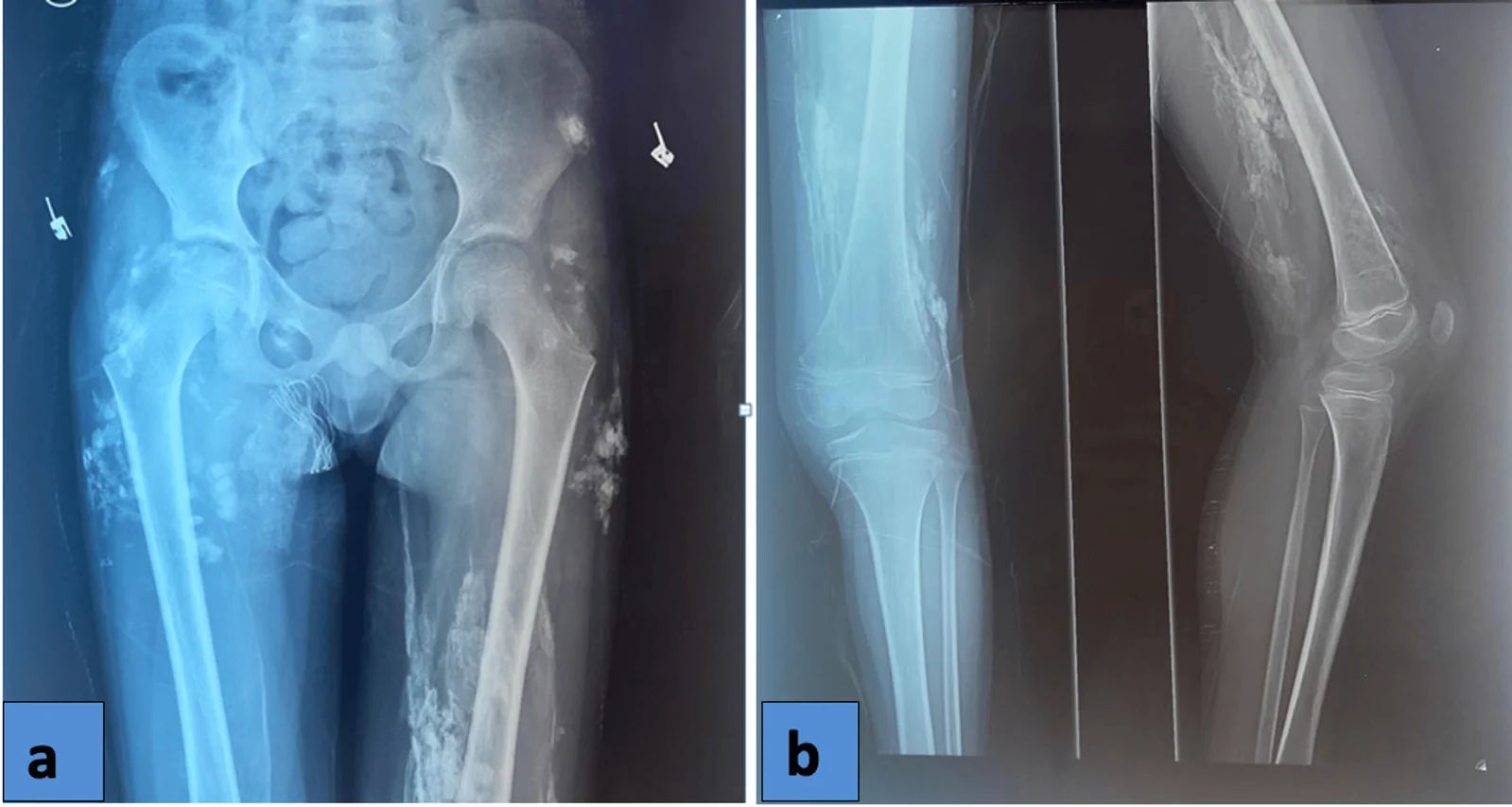
Plain X-ray before ESWT treatment Plain X-ray of the pelvis (a) and of the knee (b) ESWT: extracorporeal shock wave therapy

On admission to the PRM department, 14 calcifications were found in different areas of the body (Table 1). The surface areas of the calcifications ranged from 0.5 to 9.0 cm2. The complaint of pain is in the largest area in the gluteal region, rated 7/10 on the numerical pain scale, and the other areas are painless. His pain and the functional limitation of his knee resulted in gait disturbances with a loss of self-confidence and major repercussions on his school attendance and participation. The patient received 8 sessions of ESWT (Figure 3) at 1-week intervals with a protocol of 1,000 pulses and 10 HZ frequency for each calcinosis. The sessions were well-tolerated, without adverse events. The areas of calcinosis were assessed clinically by palpation and measured using a ruler. These measurements, the numerical pain score, and the radiological assessment were obtained before the first ESWT session and at the 24-month follow-up. Radiographic assessment was qualitative and did not include quantitative imaging measurements.

**Table 1:** Topography and clinically measured area of calcinosis before and 24 months after ESWT treatment ESWT: extracorporeal shock wave therapy

Topography of calcinosis	Area cm2	
	Before ESWT	After ESWT
Upper limbs		
Side of the right arm	0,5	0
Side of the left arm	0,5	0
Inside of the left arm	1,5	1,5
Lower limbs		
Right upper external gluteal quadrant	9	7,5
Posterior aspect of the right thigh (upper third)	1,5	1
Posterior aspect of the right thigh (middle third)	1	1
Left upper outer gluteal quadrant	1	0,25
Right popliteal fossa	0,75	0
Posterior aspect of the left thigh (middle third)	3	2
Posterior aspect of the right thigh (lower third)	6	3,75
Lateral aspect of the left thigh	0,5	0
Opposite the greater trochanter	0,5	0
Lateral aspect of the left thigh	1	0,5
Lateral aspect of the left thigh	0,75	0

**Figure 3:**
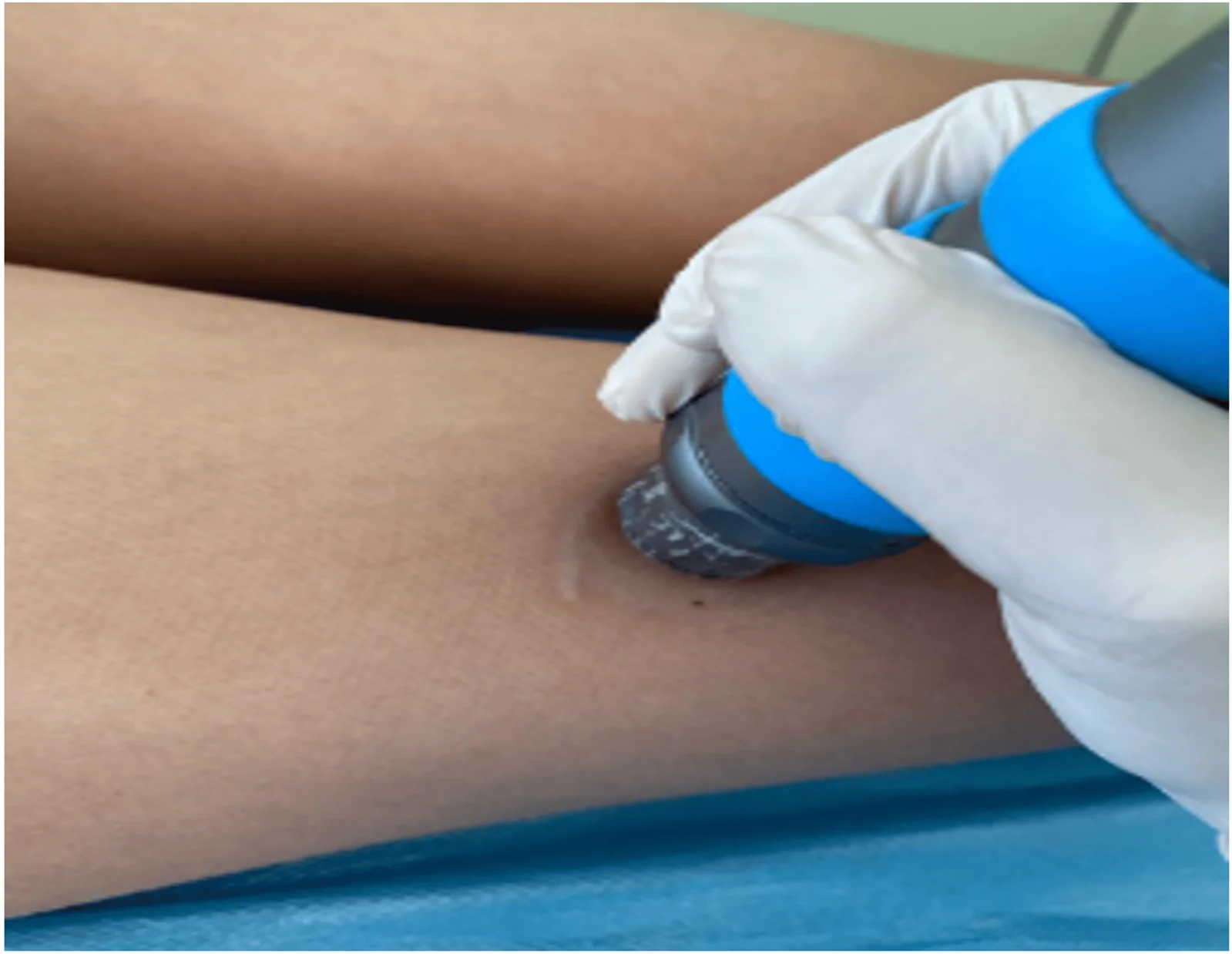
Application of ESWT to the calcified area of the posterior thigh ESWT: extracorporeal shock wave therapy

The outcome was favorable from both the clinical (Figure 4) and radiological perspectives (Figure 5 ). The evolution 24 months after the last ESWT treatment session was marked by the complete disappearance of all the subcentimetric zones and the zones on the lateral aspect of the left arm and the right popliteal fossa, and a decrease in the surface area of several zones. In addition, the calcified area in the left arm remained stationary. Pain decreased to 3/10 in the gluteal region while all other areas remained pain-free. The range of motion of the affected knee returned to normal after disappearance of the popliteal calcinosis. This was associated with an improvement in the walking pattern with good integration into activities of daily life and a satisfactory school life.

**Figure 4:**
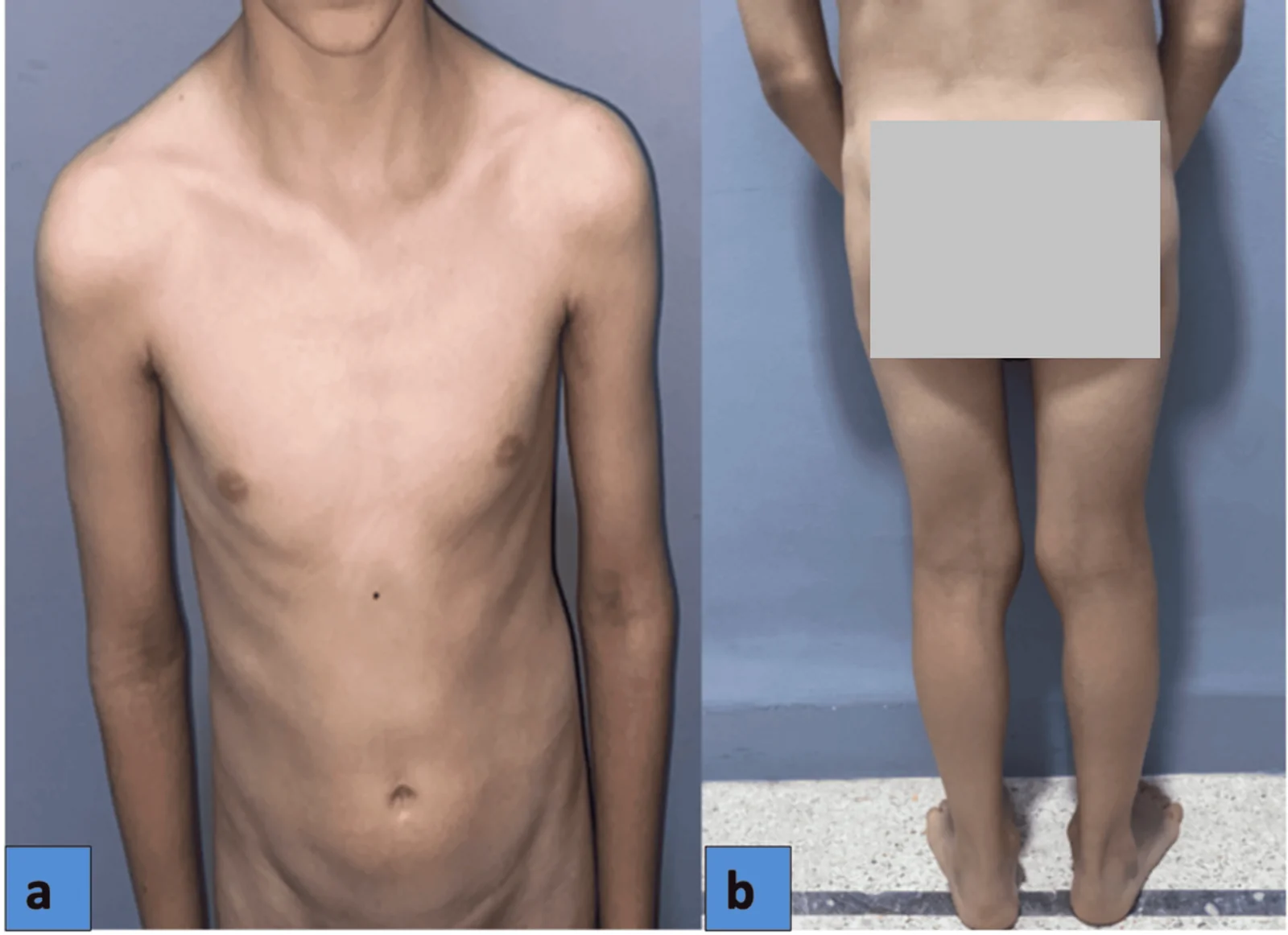
Patient’s condition after ESWT treatment Anterior trunk view (a) and posterior view of the lower limbs (b) ESWT: extracorporeal shock wave therapy

**Figure 5:**
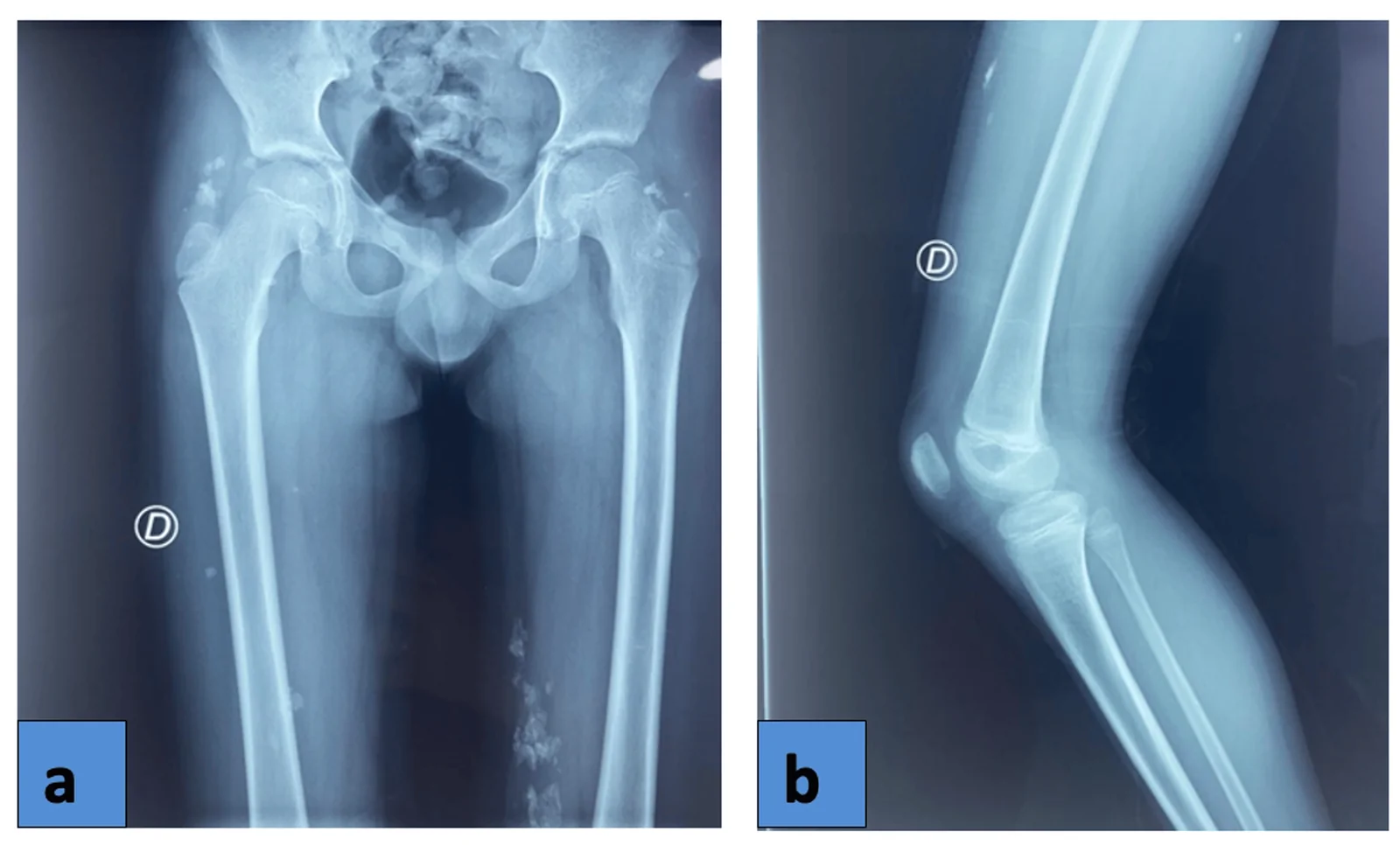
Plain X-ray after ESWT treatment Plain X-ray of the pelvis (a) and of the knee (b) ESWT: extracorporeal shock wave therapy

## Discussion

The long-term consequences of JDM include calcinosis [3], which can be responsible for complications such as ulcerations, skin infections in the first instance, and functional limitations, sometimes with a major handicap and impact on the patient's quality of life [4].

Treatment can be by drug therapy or surgery for disabling forms resistant to conservative treatment, or by non-pharmacological interventions, notably rehabilitation in case of muscle deficit or secondary joint limitation, essentially in case of calcinosis located opposite the joints; however, there is no validated reference treatment for calcinosis in dermatomyositis. Previous reports have described the use of extracorporeal shock waves for calcinosis [5], while treatment protocols may vary according to the clinical indication [6]. Evidence from rotator cuff calcific tendinopathy suggests that ESWT may improve pain and function and may promote calcific deposit resolution; however, these findings cannot be directly extrapolated to JDM-associated calcinosis [7].

Published evidence on ESWT for calcinosis cutis remains limited, with treatment protocols generally involving a small number of sessions and short follow-up periods [5]. Standardized treatment protocols and systematic radiological follow-up remain lacking.

In the current practice of the use of ESWT, the number of sessions varies between one and six; in rare cases, it is possible to carry out up to nine sessions if there is improvement as the sessions progress [6]. For our case, we decided to conduct eight sessions because there was a functional clinical improvement and reduction of pain; this improvement was noticed from the first session.

In our case, clinical improvement was observed following ESWT; however, given the single-patient design and the limitations of the assessment, the observed changes cannot be attributed solely to ESWT, and natural evolution cannot be excluded. Radiological surveillance remains important in cases of diagnostic uncertainty, deep calcinosis, and for assessing the extent and evolution of lesions, with standard radiography being the first-line imaging modality [4]. Hence, continued monitoring and multidisciplinary care are important.

The absence of intermediate clinical and radiological assessments between treatment completion and the 24-month follow-up represents a limitation of this case report. In addition, reproducibility and blinding of the radiographic assessment were not documented.

## Conclusions

Cutaneous calcinosis is a long-term complication of dermatomyositis that may lead to functional impairment and affect quality of life. Several therapeutic approaches have been proposed, but no specific treatment has been established. In our case, extracorporeal shock wave therapy, used as an adjuvant treatment, was associated with a reduction in calcinosis lesions and pain, as well as functional improvement. These observations need to be confirmed by further studies.
